# Occurrence of *Cryptosporidium* oocysts in commercial oysters in southern Thailand

**DOI:** 10.1016/j.fawpar.2023.e00205

**Published:** 2023-07-27

**Authors:** Mayuna Srisuphanunt, Polrat Wilairatana, Nateelak Kooltheat, Thanis Damrongwatanapokin, Panagiotis Karanis

**Affiliations:** a Department of Medical Technology, School of Allied Health Sciences, Walailak University, Nakhon Si Thammarat 80160, Thailand; b Department of Clinical Tropical Medicine, Faculty of Tropical Medicine, Mahidol University, Bangkok 10400, Thailand; c Akkhraratchakumari Veterinary College, Walailak University, Nakhon Si Thammarat 80160, Thailand; d University of Nicosia Medical School, Department of Basic and Clinical Sciences, Egkomi 2408, Cyprus; e Centre for One Health, Walailak University, Nakhon Si Thammarat 80161, Thailand; f Hematology and Transfusion Science Research Center, School of Allied Health Sciences, Walailak University, Nakhon Si Thammarat 80160, Thailand

**Keywords:** *Cryptosporidium*, Detection, Oysters, Coastal gulf, Thailand

## Abstract

The enteric parasite *Cryptosporidium* is spread through the fecal-oral pathway, most commonly by the consumption of contaminated water but also through food. Because eating raw or barely cooked shellfish might put consumers at risk for cryptosporidiosis, identifying the parasite in oysters is important for public health. A total of 240 oysters, collected from two shellfish aquaculture sites in Thailand's Gulf coast, Nakhon Si Thammarat and Surat Thani, were tested for the presence of *Cryptosporidium*. *Escherichia coli*, enterococci, and thermotolerant coliform total levels were measured to assess seawater quality in the shellfish production regions. Oocysts of *Cryptosporidium* spp. were detected in 13.8% of the samples processed by immunofluorescence analyses. The detection of *Cryptosporidium* spp. oocysts in oysters obtained from Surat Thani (17.5%) was higher than in those obtained from Nakhon Si Thammarat (9.2%). The difference in detection of positive samples obtained from Nakhon Si Thammarat and those obtained from Surat Thani may be attributed to the effects of physical, ecological, and anthropogenic conditions, resulting in an increased level of marine water contamination by *Cryptosporidium* spp. oocysts. These findings demonstrate that native commercial oysters obtained from Thailand's southern Gulf coast contained *Cryptosporidium* spp. oocysts which might serve as a source of human infection. Consequently, these findings pose a serious public health concern and suggest that more quality control measures need to be implemented by the oyster aquaculture business to ensure the safety of seafood.

## 1 Introduction

*Cryptosporidium* is a protozoan parasite that infects mainly the gastrointestinal (GI) tract of humans and other animals. In humans it causes widespread gastroenteritis, which is normally self-limiting but may be fatal in immunocompromised people (Srisuphanunt et al., 2008). *Cryptosporidium* is spread via the fecal-oral route through food and drink (Costa et al., 2022; Lalonde and Gajadhar, 2016; Patanasatienkul et al., 2020). *Cryptosporidium* oocysts, ubiquitous in the aquatic environment, are secreted in feces from infected hosts and are responsible for the transmissive stage. Due to their resistance to environmental stresses, they cannot be completely removed by conventional water treatment practices (Fradette et al., 2022; Franceschelli et al., 2022; Karanis et al., 2007; Merks et al., 2023).

Edible bivalve molluscs, including oysters, mussels and cockles, filter vast amounts of water and consequently acquire, retain, and concentrate pathogenic organisms such as protozoa, bacteria and viruses (Potasman et al., 2002; Wittman and Flick, 1995). Therefore, eating raw or undercooked shellfish poses a health risk to humans. *Cryptosporidium* spp. have been detected in water samples obtained from groundwater, lakes, rivers, estuaries, sewage, and the ocean (Chique et al., 2020; Fayer, 2004; Hamilton et al., 2018; Neto et al., 2006). Oocysts, the infectious form of the parasite, may contaminate molluscan shellfish harvesting sites and, consequently, humans may get infected by eating shellfish or by participating in recreational activities in these places. Several studies have found *Cryptosporidium* oocysts in mussels, clams, oysters, and cockles from various coastal regions (DeMone et al., 2020; Fayer et al., 1999; Giangaspero et al., 2005; Gomez-Bautista et al., 2000; Gómez-Couso et al., 2004; Graczyk et al., 1997). *Cryptosporidium* oocysts were also discovered in commercial shellfish obtained from 64.9% of the locations studied along the Atlantic coast of North America and Canada, either by microscopy utilizing a direct immunofluorescence assay or by molecular testing (Fayer, 2004).

The detection of these parasites in shellfish is concerning owing to the risk of developing cryptosporidiosis by eating raw or undercooked seafood. Evidence of *Cryptosporidium* transmission by shellfish intake has been observed in Thailand's central region, including Bangkok and adjacent provinces (Srisuphanunt et al., 2010). However, further studies on natural contamination by harmful protozoa are needed, especially in southern Thailand. The identification of these protozoa in shellfish is crucial given that the presence of *Cryptosporidium* oocysts and *Giardia* cysts has previously been documented, using the direct immunofluorescence antibody (IFA) tests, in ambient waters off Thailand's southwest coast (Srisuphanunt et al., 2010). IFA tests cannot discriminate between *Cryptosporidium* species and therefore molecular analysis is recommended.

Furthermore, throughout the last 20 years, shellfish output in Thailand has fluctuated between 50,000 and 300,000 metric tonnes (mt), averaging approximately 120,000 mt/yr. According to statistics from Thailand's Department of Fisheries, and the Ministry of Agriculture and Cooperatives, cultural practices account for 80% of green mussel, cockle, and oyster output (WorldFish, 2022). Given the widespread cultivation and consumption of shellfish, the aim of the present study is to assess the natural occurrence of *Cryptosporidium* contamination in commercial oysters commonly consumed in Thailand as well as to verify the microbiological quality of the seawater where they were harvested for human consumption.

## 2 Materials and methods

### 2.1 Oyster sampling sites

**Fig. 1:**
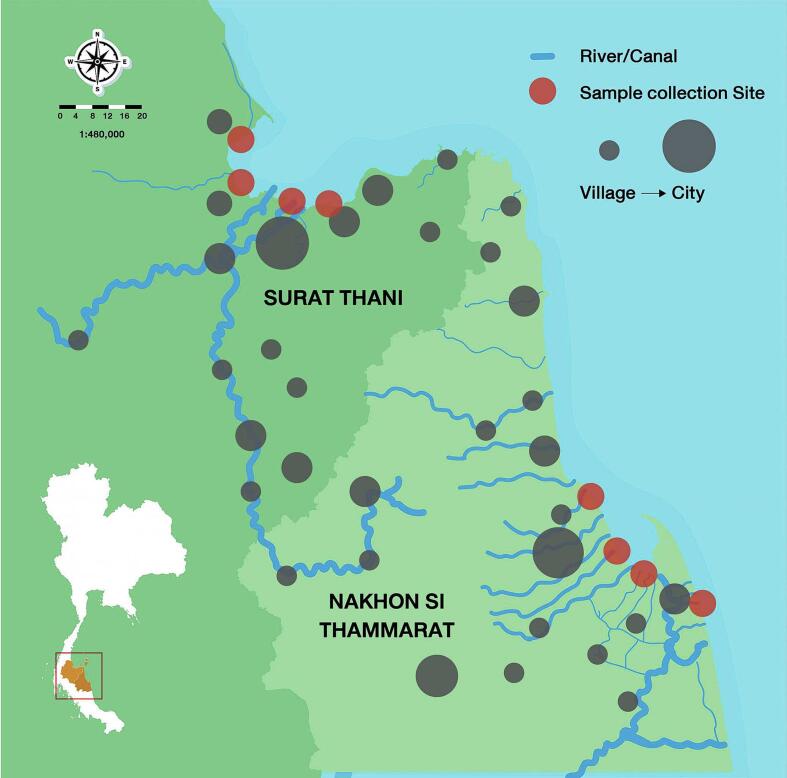
Map of selected sampling areas along the Gulf coast of southern Thailand. This study set oyster sampling locations near the mouths of major rivers in Surat Thani and Nakhon Si Thammarat. Cities and villages along the river basin produce sewage contamination that flows via the river to the sea, where oysters are cultivated. Map symbols: rivers and canals (blue lines); cities and villages (grey circles); oyster sampling sites (red circles). (For interpretation of the references to colour in this figure legend, the reader is referred to the web version of this article.)

The Bandon Bay oyster aquaculture site in Surat Thani (coordinates: 9°17′N, 99°17′E) and the Pak Phanang district of Nakhon Si Thammarat, at the mouth of the Pak Phanang River protected from the open sea by a long peninsula (coordinates: 8°31′N, 100°13′E), were chosen for the current study. This research area has several locations of household sewage discharge along the coast, an estuarine environment at the junction of the Tapi and Phum Duang Rivers, saltwater, and a commercial harvesting area (Fig. 1). Nakhon Si Thammarat has 225 km of shoreline on the Gulf of Thailand. Mueang Nakhon Si Thammarat, the capital, is a low-lying city 15 km inland, northwest of Pak Nakhon Bay. The city's water resources include a vast network of canals and streams that extend beyond the municipal limits into the adjacent rural regions. The Tha Sak, Bang Luang, Na Muang, Pa Lao, Suan Luang, and Khu Pai Canals are among the many that flow through the city's commercial and residential areas. Several offshoot rivers, including the Plai Bang Khwai, Pak Nakhon, Pak Phanang, and Tha Sak, eventually discharge into the Gulf of Thailand.

Following an intense flood season in 1988, massive canal networks in southern Thailand were initially created. Heavy losses of life, property, and agricultural land that year prompted a series of infrastructural upgrades aimed at managing flood risk and water supplies across the area. The Office of Natural Resources and the Royal Irrigation Department have significantly invested in infrastructure over the past 50 years, completing nearly 50 projects since 1974 (Office of the Royal Development Projects Board, 2020). In 2018, a large flood mitigation project was completed, which included the construction of new diversion channels and floodgates to regulate runoff.

The Tapi River, which flows 230 km from the Khao Luang Mountains via Surat Thani, and the Pak Phanang River, which runs 156 km from the Khao Bantad Mountains into Pak Nakhon Bay, are two more notable rivers in Nakhon Si Thammarat Province. According to Areepipatkul et al. (2014) urban floods and the deterioration of water quality (due to agricultural pollution, municipal waste, and wastewater) are major environmental concerns for Nakhon Si Thammarat. Flooding incidents, induced by heavy rain or tropical storms, occur daily and have been reported from 2016 to 2019 (FloodList, 2022). These environmental stresses often interact in areas where heavy flood flow sweeps garbage from land into neighbouring waters and the ocean.

*Crassostrea belcheri*, *Crassostrea lugubris,* and *Saccostrea forskali*, Thailand's most common and edible oyster species, were harvested for this study using hand tongs. Between June and November 2021, 240 oysters were collected from eight places, (5 per sample site every month). Because the oysters dwelt near sewage discharge sites, sample locations at the river mouth were selected with care. The oysters were carried refrigerated to the B8 Laboratory, Department of Medical Technology, School of Allied Health Sciences, University of Walailak, southern Thailand, for analysis.

### 2.2 Oyster processing

The oysters were shucked in the laboratory, following which parasitological investigation was performed as described by Guiguet Leal et al. (2008) and Srisuphanunt et al. (2009). The gills and GI tracts of twelve oysters were separated and homogenized in a tissue homogenizer with distilled water and 0.1% Tween 80 in a 2:1 ratio. The tissue pools were homogenized, and lipids were extracted using diethyl ether. After vigorously shaking the tube for 30 s, the sample was centrifuged at 1250 ×*g* for 5 min in phosphate-buffered saline (PBS 0.04 M pH 7.2). The supernatant was discarded, and the pellet was re-suspended in a 1.5 mL microcentrifuge tube with 100 μL of distilled water.

### 2.3 Detection of *Cryptosporidium* oocysts

FITC-labelled anti-*Cryptosporidium parvum* monoclonal antibodies (MeriFluor® *Cryptosporidium*; Meridian Bioscience, Inc., Cincinnati, OH, USA) were used in a direct IFA test to detect *Cryptosporidium* oocysts in oyster GI tract pellets. The oyster pellet was mixed by vortexing, and 20 μL of it was pipetted onto glass slides. The slides were air-dried for 10 min fixed with methanol and stained with antibodies. The nucleus of the cell was stained with 4′,6-diamino-2-phenylindole (DAPI). Cells were stained for 30 min in a humidified chamber, washed with PBS, and observed using differential interference contrast and fluorescence microscopy (Olympus BX51/BH2, Evident, Tokyo, JP) according to Method 1623 (United States Environmental Protection Agency, 2005; Srisuphanunt et al. (2009), 2010). The size (4–6 mm), morphology, and immunofluorescence staining results of *C. parvum* oocysts (i.e., the intense apple green fluorescence of the oocyst wall) were utilized to identify whether specimens were positive for oocysts. DAPI nucleic acid stain fluoresces blue and shows the location and number of nuclei in the oocyst. Phase-contrast microscopy was used for a complete diagnosis and validation of the parasite's appearance. To calculate the number of oocysts detected in each naturally infected oyster, the total number of oocysts identified in the examined aliquots was multiplied by the quantity of tissue homogenate and divided by the number of shellfish analyzed. The total number of oocysts per 100 μL pellet of each oyster sample was calculated by multiplying the number of oocysts observed on each colored sample slide by five.

### 2.4 Bacterial analysis

Monthly seawater samples (100 mL) were collected in sterile containers from each oyster sampling location according to the Standard Methods for the Examination of Water and Wastewater Baird and Bridgewater (2017) The most probable number (MPN) was used to count thermotolerant coliforms, *Escherichia coli*, and enterococci in seawater samples (Eaton et al., 2005). The National Environmental Quality Act (No. 2), B.E. 2561 (2018), is Thailand's federal legislation resolution that addresses environmental concerns. As per this act, seawater quality is classified as either appropriate or inappropriate for recreational usage (Ministry of Natural Resources and Environment, 2018). If any of the following indicators are confirmed, the water is considered inappropriate for use presence of >2500 thermotolerant coliforms per 100 mL, >2000 *E. coli* per 100 mL, and > 400 enterococci per 100 mL.

## 3 Results

240 oysters from eight different aquaculture farms in two regions were analyzed. *Cryptosporidium* oocysts were detected in both oyster culture sites under natural circumstances (Table 1). A total of 120 samples were analyzed from four oyster farms in Ban Don Bay, Surat Thani, among which 21 positive samples (17.5%) were identified, with a median *Cryptosporidium* oocyst count of 13 per oyster. In Nakhon Si Thammarat, 11 positive samples (9.2%) were discovered from 120 samples collected from four oyster farms in Pak Phanang Bay, with a median *Cryptosporidium* oocyst count of 9 per oyster.

**Table 1:** *Cryptosporidium* contamination in oysters from Surat Thani and Nakhon Si Thammarat along the southern gulf coast of Thailand.

Origin	No. of samples analyzed	No. of positive samples (%)	No. of oocyst/oyster in the positive sample (median)
Samples from Surat Thani			
Bandon Bay oyster culture sites	120	21 (17.5)	13
Samples from Nakhon Si Thammarat			
Pak Phanang Bay oyster culture sites	120	11 (9.17)	9
Total	240	32 (13.33)	22

The saltwater had a high concentration of microbiological indicators, and the majority of samples did not meet the acceptable legal requirement. Thermotolerant coliforms were identified in concentrations ranging from 0.9 to 3.6 × 10^4^ MPN per 100 mL of seawater; *E. coli* was detected in concentrations ranging from 4.0 to 10^3^ MPN per 100 mL of saltwater, and enterococci were detected in concentrations ranging from 2.0 to 4.7 × 10^3^ MPN per 100 mL of saltwater. The maximum contamination was detected in October 2021, when thermotolerant coliforms and *E. coli* reached levels of 3.6 × 10^4^ and 4.0 × 10^3^ MPN per 100 mL, respectively (Table 2), both of which exceeded the allowable threshold for various microbiological indicators. The microbiological data from seawater revealed that the water quality at the oyster harvesting location was subpar.

**Table 2:** Bacterial Contamination in seawater from Surat Thani and Nakhon Si Thammarat along the southern Gulf coast of Thailand.

Contaminated Bacteria	Most Probable Number (MPN)/100 mL Seawater					
	JUN	JUL	AUG	SEP	OCT	NOV
Thermotolerant coliforms (×10^4^)	3.0	3.0	3.3	3.3	3.6	3.5
*Escherichia coli* (×10^3^)	4.0	4.0	4.0	4.0	4.0	4.0
Enterococci (×10^3^)	4.0	4.0	4.5	4.7	4.7	4.5

## 4 Discussion

Diseases caused by eating shellfish have been increasing worldwide. Several epidemics associated with eating bivalve foods have been documented (Bigot-Clivot et al., 2022; DeMone et al., 2020; Géba et al., 2021; Ligda et al., 2020; Manore et al., 2020; Potasman et al., 2002; Santos et al., 2018; Willis et al., 2014).

Previous studies have shown that *Cryptosporidium* oocysts contaminate seafood in North America and Brazil (Fayer, 2004; Guiguet Leal et al., 2008; Merks et al., 2023; Miller et al., 2005), and similar findings have been reported in Europe (Gomez-Bautista et al., 2000; Schets et al., 2007). The present study is the first to report the detection of *Cryptosporidium* oocysts in bivalve mollusc oysters in Thailand. This protozoan parasite may accumulate extensively in mollusc tissues (Fayer et al., 1999; Freire-Santos et al., 2001; Graczyk and Fried, 2007). Furthermore, other studies have indicated that *Cryptosporidium* oocysts can survive in marine environments for a few days to a year (Freire-Santos et al., 2000; Tamburrini and Pozio, 1999), and their continued presence in the environment may increase the likelihood that molluscs will filter out and concentrate oocysts, which could be harmful to oyster consumers (Merks et al., 2023). Surat Thani and Nakhon Si Thammarat collect and filter about 36.0% of domestic sewage. The preferential discharge of effluents into rivers pollutes and devalues the water as it passes through ecosystems (Pollution Control Department - Ministry of Natural Resources and Environment, 2021).

The GI tract homogenates investigated in this study were more difficult to evaluate than gill homogenates owing to the presence of numerous layers on the sample slides, which may explain the lack of *Cryptosporidium* oocysts in this kind of tissue. Consequently, masking may have precluded oocyst identification in the GI system. Furthermore, Graczyk et al., 1997, Graczyk et al., 2001 demonstrated that *C.parvum* oocysts were internalized by hemocyte monolayers in native oysters (*Crassostrea virginia*), and they discovered a loss of fluorescence in the oocyst wall as contact time increased. Because the widths of pores in the gills and oocysts are comparable, the oocysts may be retained predominantly by the gills.

According to our study's findings, a median *Cryptosporidium* oocyst count of 13 per oyster in Ban Don Bay area and 9 per oyster in Nakhon Si Thannarat, if the oocysts were viable and proven to be infectious in humans, there is a significant likelihood that the consumption of these oysters would result in a *Cryptosporidium* infection especially since the infectious dose of *C. parvum* is typically 10 oocysts (Cooperative Research Centre for Water Quality and Treatment, 2008). Furthermore, it is hypothesized that people with immunological deficiencies may acquire an infection after swallowing a single oocyst, therefore putting immunocompromised populations at risk (Rose et al., 2002).

The increased level of fecal indicators, which revealed a large amount of fecal contamination at our sampling sites, may explain the high number of positive bivalves harboring *Cryptosporidium* oocysts. However, several other studies have reported no link between the quantity of fecal indicator coliforms in water and *Cryptosporidium* (Gómez-Couso et al., 2004; Gómez-Couso et al., 2003; Graczyk and Fried, 2007; Schets et al., 2007).

Different types of shellfish have been identified as biological indicators of animal and human fecal contamination in the environments in which they were discovered or grown (Giangaspero et al., 2005); in particular, alkalinity has been reported to influence oyster density in Bandon Bay, followed by salinity, pH, NO_2,_ and NH_4_N_2_ (Chumkiew et al., 2015). This is consistent with our study's results that the prevalence of *Cryptosporidium* oocysts was greater in Surat Thani than in Nakhon Si Thammarat. *Saccostrea forskali* oysters, one of the three edible species harvested for this study, have emerged as a novel species capable of efficiently eliminating waterborne infections from polluted environments and must be added to this extensive list of biological indicators (Bussarawit and Cedhagen, 2012).

Notably, oyster fishing is an exploratory activity in Bandon Bay, with significant oyster aquaculture sites in Surat Thani and Nakhon Si Thammarat's Pak Phanang District, where the local population harvest large numbers for subsistence with no oversight from federal organizations or capable environmental agencies. Because Thailand's Federal Legislation-Central Environmental Authority decision only permits the examination of fecal coliforms in waterways where molluscs are grown, monitoring this protozoan is critical (Pollution Control Department - Ministry of Natural Resources and Environment, 2021).

## 5 Conclusion

*Cryptosporidium* was detected in oysters obtained from Thailand's southern Gulf coast. Consuming raw shellfish, especially oysters, may contribute to the spread of food-borne infections and pose a life-threatening hazard to humans, particularly immuno-compromised individuals. Although the species of *Cryptosporidium* detected in the oysters remains unknown, it is important for determining the risk posed to people. People with impaired immune systems in developed countries are more aware of the hazards of such infections and thus avoid consuming raw foods. However, this is not the case for Thailand's poorest residents. Our report offers crucial information and should encourage local health professionals to implement a shellfish screening program for *Cryptosporidium* oocysts. Future studies should focus on viability of *Cryptosporidium* infecting the oysters, identifying the species by molecular analysis, detecting this parasite in saltwater and establishing how it interacts with salinity, pH, and temperature.

## Funding

This work was supported by 10.13039/501100010034Walailak University, Thailand (Grant # WU-IRG-64-077).

## Declaration of Competing Interest

The authors declare no conflicts of interest.
